# Low risk of OHSS with follitropin delta use in women with different polycystic ovary syndrome phenotypes: a retrospective case series

**DOI:** 10.1186/s13048-021-00773-5

**Published:** 2021-02-12

**Authors:** Stephanie Yacoub, Kenneth Cadesky, Robert F. Casper

**Affiliations:** 1 Present address: Department of Obstetrics and Gynecology, Saint Georges Hospital University Medical Center, Beirut, Lebanon; 2 TRIO Fertility, and Department of Obstetrics and Gynecology, University of Toronto, 655 Bay St, Suite 1101, Toronto, ON M5G2K4 Canada; 3 Lunenfeld-Tanenbaum Research Institute, Mount Sinai System, Toronto, ON Canada

**Keywords:** Polycystic ovary syndrome, Rotterdam criteria, Follitropin delta, Recombinant FSH, OHSS

## Abstract

**Background:**

To explore the efficacy of follitropin delta in ovarian stimulation of patients with the Rotterdam ESHRE/ASRM 2003 phenotypes of polycystic ovarian syndrome (PCOS) using a retrospective case series with an electronic file search in a reproductive medicine clinic.

**Case presentation:**

Seventy-four patients with PCOS undergoing ovarian stimulation according to the individualized dosing algorithm of follitropin delta for in vitro fertilization/intracytoplasmic sperm injection (IVF/ICSI)/oocyte freezing were included. Follitropin delta resulted in a high number of pre-ovulatory follicles at the end of stimulation as expected in patients with PCOS. There was a large number of oocytes retrieved with an acceptable percentage of metaphase II (MII) oocytes. There were no cases of moderate or severe OHSS across all phenotypes.

**Conclusion:**

Follitropin delta, using the individualized dosing algorithm, appears to be a safe method of ovarian stimulation with a low risk of OHSS in PCOS patients without sacrificing successful stimulation outcomes.

## Background

Polycystic ovary syndrome (PCOS) encompasses a spectrum of reproductive and metabolic abnormalities [5] with a prevalence between 4 and 21% [14]. The commonly used Rotterdam classification, set in 2003 by the European Society for Human Reproduction and Embryology and the American Society for Reproductive Medicine, divides PCOS into four main phenotypes based on features of clinical and or biochemical signs of hyperandrogenism (HA), ovulatory dysfunction (OD) and polycystic ovary morphology (PCOM) [7, 14, 18], highlighted in Table 1.

**Table 1:** Rotterdam ESHRE/ASRM 2003 criteria for categorization of the different phenotypes of PCOS

Overall PCOS Diagnosis (Revised 2003 consensus on diagnostic criteria and long term health risks related to polycystic ovary syndrome, 2004)	Phenotype [18]	
Diagnosis confirmed by 2 of 3 following criteria after exclusion of other etiologies:	A	HA, OD & PCOM
	B	HA & OD
Hyper-androgenism (HA) Ovulatory disorder (OD) Polycystic ovarian morphology (PCOM)	C	HA & PCOM
	D	OD & PCOM

Women with PCOS have a high incidence of ovarian hyperstimulation syndrome (OHSS) due to the large cohort of follicles that can be recruited with gonadotropin therapy [11, 16]. This applies particularly to women with phenotypes A and B who have the highest anti-Mullerian hormone (AMH) levels [10].

OHSS is an iatrogenic complication of ovarian stimulation that is potentially life threatening [8]. A challenge for fertility specialists is prevention and finding a balance between ovarian stimulation for a successful outcome and reducing the patient’s risk of OHSS [8, 11]. As a result, certain characteristics and diagnostic markers of a patient’s ovarian reserve, namely AMH, are used to identify the patients at risk of developing OHSS and individualize the starting dose of gonadotropin [8, 15]. However, with conventional highly purified urinary gonadotropins or with recombinant follicle stimulating hormone (FSH or follitropin) alpha, clinicians must make an educated guess as to a starting dose that will produce an optimal number of oocytes while reducing the risk of OHSS.

Follitropin delta is a new recombinant FSH that is uniquely administered according to an individualized dosing algorithm including patient’s body weight and pretreatment AMH level with the goal of reducing risks of OHSS [2, 8, 12, 15]. Several published trials have established that follitropin delta has a similar safety profile and is non inferior with regards to ongoing pregnancy rates when compared to the more conventional gonadotropin, follitropin alfa [2, 8, 15].

No information is available in the literature regarding the outcomes of the use of follitropin delta for ovarian stimulation in regard to women who exhibit the different Rotterdam 2003 phenotypes of PCOS. In this study, the aim was to assess characteristics and responses of patients with different PCOS phenotypes when follitropin delta was used for ovarian stimulation with special focus on occurrence of OHSS.

## Case presentation

This study was a retrospective case series of patients who were treated with follitropin delta (Rekovelle, Ferring Pharmaceuticals) for ovarian stimulation at Trio Fertility in Toronto, Ontario involving an electronic search of IVF charts of patients between February 2016 and March 2020.

Patient files included the standard investigation: the medical history, physical exam: including weight, height, antral follicle count (AFC) and AMH level. Sonohysterogram explored the patient’s uterine cavity. Also included was a detailed male partner history, physical exam and semen analysis interpreted according to the World Health Organization (WHO) criteria.

All patients who were administered follitropin delta for ovarian stimulation were included, regardless of age, body mass index (BMI), menstrual cycle duration, or AMH levels. All women were Caucasian. Once patients who received follitropin delta were identified from the electronic medical record, their data was anonymized for subsequent analysis and manuscript preparation and the authors were unaware of any identifying information. Exclusion criteria comprised missing data for the cycle in which follitropin delta was prescribed and also patients who did not meet the criteria for PCOS according to the 2003 Rotterdam consensus.

Follitropin delta was administered by a daily subcutaneous dose determined by AMH level and body weight before initiating treatment. AMH < 15 pmol/L: 12 μg; AMH ≥ 15 pmol/L: 0.10–0.19 μg/kg; maximum daily dose of 12 μg, shown in Table 2.

**Table 2:** Dosing algorithm for follitropin delta based on AMH level [12]

AMH (pmol/L)	< 15	15–16	17	18	19–20	21–22	23–24	25–27	28–32	33–39	≥ 40
3Daily dose (μg)	12 (μg)	0.19 (μg/kg)	0.18 (μg/kg)	0.17 (μg/kg)	0.16 (μg/kg)	0.15 (μg/kg)	0.14 (μg/kg)	0.13 (μg/kg)	0.12 (μg/kg)	0.11 (μg/kg)	0.10 (μg/kg)

Seventy-two patients in this study were administered a gonadotropin releasing hormone (GnRH) antagonist while two patients received a long agonist protocol. The cycles were monitored by serum hormone levels and transvaginal ultrasound to record follicular size and number as well as endometrial thickness.

There were three methods of final triggering of oocyte maturation: human chorionic gonadotropin (hCG), GnRH agonist, or GnRH agonist and hCG (double trigger) which were administered 36 ± 2 h before oocyte pick-up. The retrieved oocytes then underwent fertilization by IVF/ ICSI with either fresh embryo transfer or freezing for frozen embryo transfer, or oocyte freezing, a plan that had been reviewed with each patient. For this study, a clinical pregnancy was defined as having a positive serum hCG and ultrasound confirmation of a positive fetal heart rate.

**Fig. 1:**
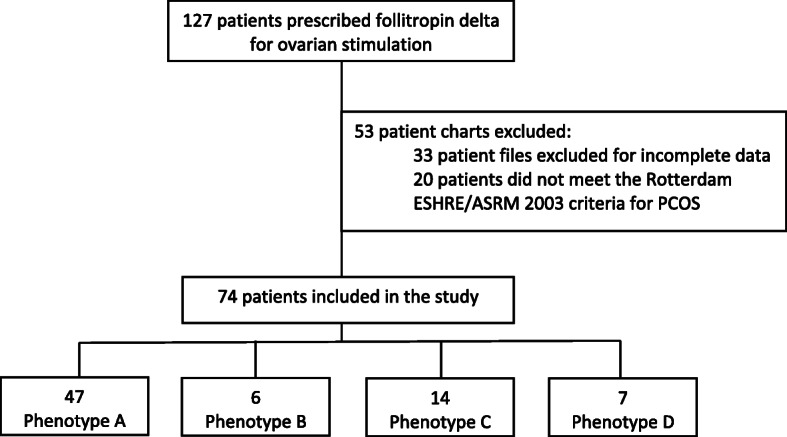
Participant flow and classification according to PCOS phenotype

There were 127 patients who were prescribed follitropin delta for ovarian stimulation during the study period. Thirty three patient files were excluded either because the treatment algorithm was not used or because the files were missing certain key data required for this study. Also, twenty of the remaining patients did not meet the Rotterdam 2003 criteria for PCOS. This resulted in 74 patients eligible for this case series who were treated with follitropin delta for ovarian stimulation according to the algorithm. These patients were then categorized according to the phenotypes mentioned above; 47 women exhibited PCOS of Phenotype A, 6 patients of Phenotype B, 14 of C and 7 of D, shown in the participant flow in Fig. 1.

Table 3 displays the patient demographics according to each phenotype of PCOS. Patients; ages at cycle initiation were quite similar across the four phenotypes. The largest proportion - 63.5% - of patients included belonged to phenotype A which contained the highest body weights and BMI. The AMH levels and AFC were higher in phenotypes A and C compared to B and D. Approximately 40–50% of patients in each phenotype (except for phenotype C where it was 14%) had been prescribed supplementary treatments for infertility prior to initiation of ovarian stimulation including lifestyle modifications with a healthy diet, physical activity, acupuncture and medications including metformin and supplements such as coenzyme Q10.

**Table 3:** Patient demographics and baseline characteristics of the four phenotypes of PCOS

	Phenotype A	Phenotype B	Phenotype C	Phenotype D
Total number of patients (*n)*	47	6	14	7
Age (*years)*	34.3 (25–49)	38.9 (36–40)	32.7 (27–43)	34.2 (31–38)
Body Weight (*kg)*	75.5 (46.7–117.0)	66.9 (54.4–81.6)	60.2 (51.0–104.0)	60.5 (47.0–79.4)
BMI (*kg/m*^*2*^*)*	28.8 (18.7–40.0)	24.0 (19.2–29.0)	24.3 (19.2–37.4)	22.3 (17.8–29.1)
Menstrual cycle Duration *(days)*	33.5 (16–60)	27 (23–30)	28.5 (26–32)	28.5 (26–33)
AMH level *(pmol/l)*	55.5 (2.2–131.7)	7.9 (2.7–11.4)	30.4 (5.2–82.1)	26.1 (14.4–33.8)
AFC- for both ovaries (*n)*	38.7 (1–40)	13.5 (3–19)	25 (10–40)	24 (15–30)
Average time interval between hormone analysis (AMH) and start of treatment with follitropin delta *(months)*	6.7	5.8	6	7.4
Number of patients prescribed treatments for infertility prior to ovarian stimulation (lifestyle modifications/metformin, etc) *(n)*	21	3	2	3
Number of patients on agonist protocols *(n)*	0	0	2	0
Number of patients on antagonist protocols *(n)*	47	6	12	7
Number of first IVF/ICSI cycles *(n)*	31	6	10	4
Number of subsequent IVF/ICSI cycles *(n)*	16	0	4	3

All patients had an AMH within 12 months before initiating treatment with follitropin delta. As PCOS is a major risk factor for OHSS during treatment, 97.3% of patients were stimulated with the antagonist protocol and only 2 patients in phenotype C with the GnRH agonist protocol. Across all phenotypes, the majority of patients were undergoing their first IVF or ICSI cycle.

During this study, all patients were dosed according to the algorithm with adjustments made during the cycle if needed. The majority of patients were dosed entirely according to the algorithm, particularly in phenotypes B and C. Most dose adjustments involved a decrease in the dose with only two patients in phenotype A with an increase.

The daily dose of follitropin delta administered in patients dosed according to the algorithm was highest in phenotype B with a mean daily dosage of 11.7 mcg (Table 4). The mean daily dose in phenotypes A and C was the most similar in patients dosed according to the algorithm and those with dosing deviations. All patients had a similar length of treatment averaging 10 days.

**Table 4:** Follitropin delta dosing as calculated using the algorithm based on AMH. All patients started with the calculated dose but some patients had reduction in dosing during the cycle

		Phenotype A	Phenotype B	Phenotype C	Phenotype D
Pts dosed according to algorithm	Daily dose of follitropin delta *(mcg)*	9.6 (5.6–12.0)	11.7 (10.0–12.0)	9.2 (4.6–12.0)	8.8 (5.7–12.0)
	Length of treatment (*days)*	10.5 (6.0–18.0)	10.1 (7.0–14.0)	10.1 (8.0–14.0)	10.6 (9.0–12.0)
Patients with dosing deviations during the cycle	Daily dose of follitropin delta (mcg)	8.2 (5.6–12)	0 (0)	8.3 (4.6–12)	6 (6)
	Length of treatment *(days)*	9.4 (8–11)	0 (0)	10 (9–11)	9 (9)

Trigger of ovulation was individualized using: hCG, GnRH agonist or double trigger as shown in Table 5. Preventive measures for patients at risk of developing OHSS included administration of a dopamine agonist or an aromatase inhibitor after the oocyte retrieval and a request to return for an ultrasound for monitoring of pelvic fluid levels and ovary size. Four cycles were cancelled in phenotype A and 1 case in phenotype B and 1 cycle was converted to IUI, all for the reason of poor ovarian response.

**Table 5:** Information on trigger of ovulation in each of the four PCOS phenotypes. Cancelled cycles or cycles converted to intrauterine insemination (IUI) were all related to poor response

		Phenotype A	Phenotype B	Phenotype C	Phenotype D
Triggering of final oocyte maturation	hCG	13	2	11	0
	GnRH agonist	13	3	1	4
	Double trigger	17	0	2	3
Preventive measures		15	0	7	3
No, of cancelled cycles *(n)*		4	1	0	0
No. of cycles converted to IUI due to poor response *(n)*		0	0	1	0

**Fig. 2:**
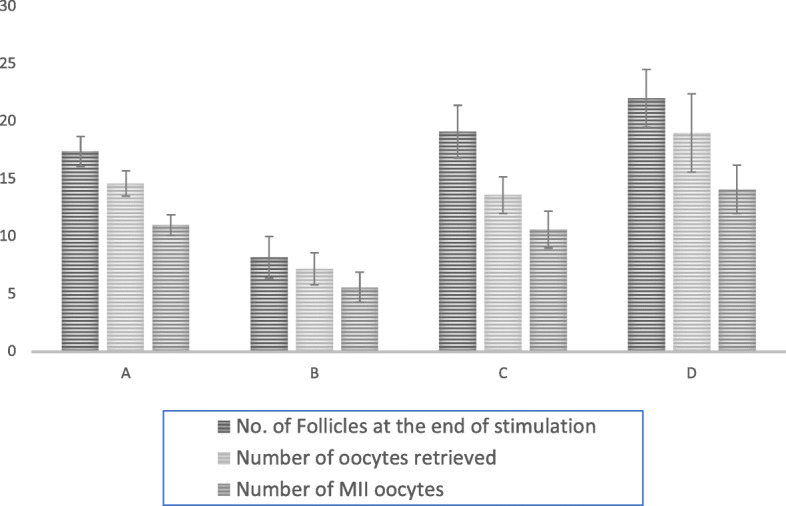
Responses to Ovarian Stimulation with Follitropin Delta According to the 4 PCOS Phenotypes (Type A,B,C and D)

Table 6 and Fig. 2 summarizes outcomes of the patients in the four phenotypes of PCOS. Phenotypes D, C and A had the greatest number of follicles at the end of stimulation as well as the highest number of oocytes retrieved and number of MII oocytes. Most patients underwent a frozen embryo transfer. OHSS occurred at a rate of 20% in patients in phenotype C, 14.3% in phenotype D and 12.7% in A. All cases of OHSS were grade 1 (abdominal distention and discomfort) and resolved without intervention. Phenotype C depicted the highest clinical pregnancy rate of 50%, followed by phenotypes D (42.8%) and A (40.4%).

**Table 6:** Ovarian response and outcomes including number (Nb) of follicles, oocytes, MII oocytes and type of embryo transfer and cases of OHSS

	Phenotype A	Phenotype B	Phenotype C	Phenotype D
Nb of follicles at the end of stimulation *(n)*	17.4 ± 1.3	8.2 ± 1.8	19.1 ± 2.3	22 ± 3.5
Nb of oocytes retrieved *(n)*	14.6 ± 1.1	7.2 ± 1.4	13.6 ± 1.6	19 ± 3.4
Nb of MII oocytes *(n)*	11 ± 0.9	5.6 ± 1.3	10.6 ± 1.6	14.1 ± 2.1
Nb of patients undergoing each type of embryo transfer *(n)*				
Fresh	5	1	1	0
Frozen	27	0	11	5
Clinical Pregnancy *(n)*	19	0	7	3
OHSS				
*n*	6	0	3	1
Grade of OHSS	(1)	–	(1)	(1)

### Statistical analysis

This was basically a descriptive case series and no statistical analysis was performed with the exception of calculating the mean +/− SEM for certain results.

## Discussion

This case series investigated ovarian stimulation with the use of follitropin delta in four phenotypes of PCOS women. The most common phenotype was A, which has also been reported in the literature to be the most prevalent phenotype [5]. Those in phenotype A exhibited the highest body weights and BMI as well as the highest AMH levels compared to the other phenotypes. Previous studies have shown that women with ‘classical’ and ‘severe’ PCOS have the highest AMH levels [17, 19].

Almost all patients were stimulated with the antagonist protocol. As per a Cochrane meta-analysis, treating patients with GnRH antagonists resulted in a lower rate of OHSS than with GnRH agonists despite no difference in clinical pregnancy rates between the two [1] and are therefore recommended to reduce the risk of OHSS [3].

According to Selcuk, patients with PCOS morphology display an easier stimulation whereas hyperandrogenemia results in better quality embryos and pregnancy rates [18]. In this study, the phenotypes with PCO morphology (phenotypes A, C and D) had the higher number of follicles formed at the end of stimulation, highest number of oocytes retrieved and almost double the number of MII oocytes compared to phenotype B. Thirty-seven patients across phenotypes chose oocyte or embryo freezing and so the clinical pregnancy rate in this study is not considered as a clinical outcome measure. Hence, the clinical pregnancy rate with follitropin delta could not be fully assessed.

The patients who are at the highest risk for OHSS are high responders, those with PCOS, a high AMH, with more than 18 oocytes retrieved and with serum estradiol concentrations > 5000 pmol/L [2, 16]. In addition, patients with a history of OHSS or elevated response to gonadotropins are also at risk [11]. According to Fischer et al., 10% of patients in all phenotypes of PCOS undergoing stimulation with follitropin alfa developed moderate and severe OHSS [9]. None of the patients stimulated with follitropin delta in this study developed grade 2 or greater OHSS [13] despite oocyte retrieval numbers up to a maximum of 37 and with some patients having more than 18 oocytes in all subgroups except Group B.

Follitropin delta resulted in absence of significant OHSS without sacrificing a good ovarian response when compared to the literature [4, 6]. The results showed a greater than average number of follicles at the end of stimulation, higher number of oocytes retrieved and higher number of MII oocytes in phenotypes A, C and D, i.e. patients with polycystic ovarian morphology.

The main limitations of this study are its retrospective nature and the small population size. There were confounding factors that could have affected the results. For example, there were three different triggers for final oocyte maturation. Also, the overall clinical pregnancy rate could not be determined since some patients did not undergo embryo transfer and opted for oocyte and embryo freezing.

A major weakness of this case study is the lack of a comparator group of matched controls resulting in the need for literature comparisons. An earlier trial demonstrated the non-inferiority of follitropin delta compared to follitropin alfa in regard to ongoing pregnancy and implantation rate in patients with ovulatory PCOS ovarian morphology. In addition, follitropin delta showed an increased predictability and a higher safety profile [11]. In the present study, there were no cases of moderate or severe OHSS in patients stimulated with follitropin delta despite high risk factors in many of the PCOS patients.

To our knowledge, this is the first study that explores the effects of follitropin delta on ovarian stimulation according to the different phenotypes of PCOS. Proper controlled trials are needed to explore the full safety profile and benefits of follitropin delta in the population of patients with PCOS.

## Data Availability

Anonymized data available on request from corresponding author.

## Abbreviations

ESHRE: European Society for Human Reproduction and endocrinology
ASRM: American Society for Reproductive Medicine
IVF: In vitro fertilization
ICSI: Intracytoplasmic sperm injection
PCOS: Polycystic ovarian syndrome
HA: Hyperandrogenism
OD: Ovulatory dysfunction
PCOM: Polycystic ovary morphology
AMH: Anti-Mullerian hormone
OHSS: Ovarian hyperstimulation syndrome
FSH: Follicle stimulating hormone
AFC: Antral follicle count
WHO: World Health Organization
BMI: Body mass index
hCG: Human chorionic gonadotropin
GnRH: Gonadotropin releasing hormone
MII: Metaphase II
